# Changes in food sufficiency among Korean adults in urban and rural areas during the COVID-19 pandemic: an analysis of the 7th and 8th Korea National Health and Nutrition Examination Survey

**DOI:** 10.4178/epih.e2024045

**Published:** 2024-04-16

**Authors:** Sarang Jeong, Jin-Young Jeong, Sohyun Park

**Affiliations:** 1 The Korean Institute of Nutrition, Hallym University, Chuncheon, Korea; 2 Hallym Research Institute of Clinical Epidemiology, Hallym University, Chuncheon, Korea; 3 Department of Food Science and Nutrition, Hallym University, Chuncheon, Korea

**Keywords:** COVID-19, Pandemics, Food security, Urban health, Rural health

## Abstract

**OBJECTIVES:**

Understanding changes in food sufficiency within various demographic groups during emergency situations, such as the global coronavirus disease 2019 (COVID-19) pandemic, is crucial in formulating public health policies for future preparedness. This study investigated potential differences between urban and rural residents in food sufficiency trends during the COVID-19 pandemic and examined how these changes varied according to socio-demographic factors.

**METHODS:**

This cross-sectional study analyzed data from 19,724 adults aged 20 years and older, utilizing information from the 7th-8th Korea National Health and Nutrition Examination Survey (2018-2021).

**RESULTS:**

In urban areas, across all subpopulations, food sufficiency improved significantly during the COVID-19 period relative to pre-pandemic levels (p<0.001). However, in rural regions, a significant increase in food sufficiency during the COVID-19 era was observed only among female, with an odds ratio of 1.42 (95% confidence interval, 1.06 to 1.89). Nevertheless, no significant interaction terms were found between region and various socio-demographic factors regarding changes in food sufficiency during the COVID-19 period.

**CONCLUSIONS:**

During the COVID-19 pandemic, food sufficiency among urban residents improved compared to the pre-pandemic era, whereas their rural counterparts saw no such improvement. Additionally, no significant interaction was detected between urban versus rural areas and changes in food sufficiency during the COVID-19 period. These findings indicate the need for targeted food policies to prepare for potential future pandemics, particularly in rural areas, where food sufficiency did not improve.

## GRAPHICAL ABSTRACT

[Fig f1-epih-46-e2024045]

## Key Message

• This study examined changes in food sufficiency among adults in urban and rural areas during the COVID-19 pandemic, focusing on socio-demographic factors.

• Findings revealed that food sufficiency significantly increased in urban areas across various demographic subgroups during the COVID-19 period, while rural areas showed no significant overall change, except for an increase among female.

• The study highlights the importance of considering socio-demographic factors and regional differences, particularly the need for targeted food assistance programs in rural areas, for future pandemic preparedness.

## INTRODUCTION

In March 2020, the World Health Organization declared coronavirus disease 2019 (COVID-19) a global pandemic [1]. Several countries, including Korea, implemented lockdown or social distancing measures to prevent the spread of the virus [2,3]. The emergence of COVID-19 has meaningfully impacted not only human life and health but also socioeconomic conditions [4-7]. The resulting decrease in household income [8] has prompted a shift in the dietary habits of low-income families towards less healthy options [9]. This change has resulted in disparities in nutrition intake and health-related behaviors during the COVID-19 period, depending on income level [10]. In Australia, the pandemic has led to decreased physical activity and poorer dietary outcomes, especially in economically vulnerable households with children aged 12 years and under [9]. Similarly, in Canada, adults aged 25 years and older have reported decreased physical activity and increased consumption of junk food and alcohol due to the pandemic, leading to adverse health effects [11]. Consequently, various studies are being conducted worldwide to investigate the changes caused by the COVID-19 pandemic in different countries.

Since the emergence of COVID-19, widespread reports have indicated increased food insufficiency due to income reduction [12]. In particular, low-income families with children have displayed heightened vulnerability [13,14]. An analysis using data from the Korea National Health and Nutrition Examination Survey (KNHANES) revealed no significant changes in food sufficiency during the COVID-19 pandemic compared to the pre-pandemic period. However, a previous study comparing the dietary habits of individuals who were and were not experiencing food insufficiency found that the group facing food insufficiency exhibited greater nutritional imbalances [15]. Thus, the pandemic may have exacerbated issues of food insufficiency and nutritional imbalances, particularly in socioeconomically disadvantaged groups. Moreover, this heightened food insufficiency substantially influences health indicators and is associated with chronic diseases such as obesity, diabetes, and hypertension [16].

Food sufficiency involves ensuring consistent access to food, adequate dietary intake, and the proper availability and utilization of nutrition [17,18]. Therefore, the assessment of food sufficiency is a crucial measure for determining whether a nation or individual can access and use safe, nutritionally adequate food [19]. Previous research has indicated variations in the proportion of the population achieving food sufficiency based on factors such as age, residential area, and personal income. Maeng et al. [20] found that individuals aged 65 years and older were less likely to experience food sufficiency compared to adults aged 19-64 years. Additionally, prior findings have indicated that food insufficiency tends to be more prevalent in rural areas than in urban regions as age increases [21]. Evidence also suggests that food insufficiency is more common among low-income households, particularly among older adults within these households [22,23].

Thus, comparing urban and rural areas regarding changes in food sufficiency during the COVID-19 pandemic, as well as understanding the relationship between these changes and sociodemographic factors, is crucial for laying the groundwork for public health policies intended for future pandemic responses. However, little research has been conducted on the regional differences in COVID-19-related changes in food sufficiency within Korea. Moreover, since urban and rural populations are not homogeneous, it is important to explore whether differences in socio-demographic characteristics contribute to distinct experiences of food sufficiency changes in each area. Therefore, this study was conducted to examine the potential for disparate changes in food sufficiency experienced by urban and rural residents before and during the COVID-19 pandemic. In addition, the study investigated whether these changes varied according to key socio-demographic factors, utilizing data from the KNHANES.

## MATERIALS AND METHODS

### Analytical data and study participants

This cross-sectional study utilized raw data from the 7th and 8th KNHANES, conducted between 2018 and 2021. A total of 21,059 individuals aged 20 years and older who participated in the KNHANES were enrolled in this study. From those who took part in the food safety survey, 19,724 individuals were ultimately selected for analysis after applying weighting procedures. Of these respondents, 10,629 participated in the 7th KNHANES (2018- 2019), which was conducted prior to the emergence of COVID-19; in comparison, 9,095 participated in the 8th KNHANES (2020- 2021) during the COVID-19 outbreak. Stratified by sex, 4,492 male and 6,137 female individuals were included before the COVID-19 outbreak, while 3,913 male and 5,182 female respondents were included during the outbreak.

### General characteristics

The analysis incorporated basic variables from the KNHANES, including sex, age, household income quartile, urban/rural classification, and education level. Age was divided into 2 categories: adults aged 20-64 years and seniors aged 65 years and older. Household income was segmented into quartiles: low, low-medium, medium-high, and high. Regional classifications were defined as “urban” for *dong* areas and “rural” for *eup/myeon* areas. Education level was categorized into 4 groups: elementary school or below, middle school, high school, and college or higher.

Data on smoking status, alcohol consumption, aerobic physical activity, and perceived stress were derived from the health survey section of the KNHANES. Smoking status was categorized as “yes” for individuals who reported smoking “more than 5 packs (100 cigarettes)” in their lifetime and “no” for those who smoked “less than 5 packs (100 cigarettes)” or had “never smoked”. Alcohol consumption was classified as “yes” for participants who reported drinking “monthly or more frequently” within the past year and “no” for those who indicated not drinking at all in the past year. Aerobic physical activity was considered “practiced” for individuals engaging in “moderate to vigorous physical activity weekly”. Stress perception was defined as “perceived” for those who reported feeling “very much” or “quite a bit” stressed.

### Anthropometric measurements

Data on weight, body mass index (BMI), and waist circumference (WC) were obtained from the health examination section of the KNHANES. To account for clothing, a correction of 0.5 kg was subtracted from the weight measurements of individuals not dressed in examination attire. For further details on these measurement methods, please refer to the 7th-8th KNHANES report [24].

### Food sufficiency

Food sufficiency was evaluated based on dietary habits data collected consistently during the 7th and 8th cycles of the KNHANES. Responses indicating that the participant “had sufficient quantity and variety of food” were classified as “sufficient.” In contrast, responses such as “had enough quantity but lacked variety in food consumption,” “occasionally lacked food due to economic difficulties,” and “frequently lacked food due to economic difficulties” were categorized as “insufficient.”

### Assessment of energy and nutrient intake

This study examined the daily nutrient intake of participants, expressed as a percentage of the Dietary Reference Intakes for Koreans (KDRIs), across a range of nutrients, including energy, carbohydrates, proteins, sodium, dietary fiber, calcium, iron, vitamin C, and vitamin E. Data were collected through the 24-hour dietary recall method as part of the KNHANES. Intake levels of sodium, dietary fiber, and vitamin E were assessed using the Adequate Intake criteria, whereas assessments for the other nutrients were based on the Estimated Average Requirement criteria. Furthermore, because the KDRI standards for carbohydrates, protein, fat, and saturated fat are not defined as absolute values, the study evaluated their consumption as a percentage of daily energy intake.

### Statistical analysis

Data analysis was performed using SAS version 9.4 (SAS Institute Inc., Cary, NC, USA). The analysis considered the complex sampling design of the KNHANES, incorporating strata (kstrata), clusters (cluster), and weights (weight). Categorical data from before and during the COVID-19 period were compared and tested by employing the PROC SURVEYFREQ procedure to conduct the complex sample Rao-Scott chi-square test. The results were presented as frequencies (n) and percentages (%). For the continuous variables, t-tests were conducted for age, BMI, and WC. To compare changes in food sufficiency between urban and rural areas and examine the interaction among socio-demographic factors, the PROC SURVEYLOGISTIC procedure was utilized. The dependent variable in this analysis was food sufficiency, and the odds ratios (ORs) for food sufficiency, based on socio-demographic factors in urban and rural areas, were analyzed after adjusting for sex, age, and household income. Interactions between the 2 variables were also incorporated in the model. Changes in nutrient intake before and during the COVID-19 by region were analyzed using a linear regression analysis after adjusting for age, sex, and household income. A p-value of less than 0.05 was considered to indicate statistical significance.

### Ethics statement

All survey protocols were approved by the Institutional Review Board of the Korea Disease Control and Prevention Agency (approval Nos. 2018-01-03-P-A, 2018-01-03-C-A, 2018-01-03-2C-A, and 2018-01-03-3C-A). All procedures were conducted in accordance with the Declaration of Helsinki.

## RESULTS

### General characteristics and food sufficiency status by region, before and during the coronavirus disease 2019 period

Table 1 presents the general characteristics of adults aged 20 years and older from urban and rural settings. No significant differences were present in age, sex, smoking status, household income, education level, aerobic exercise participation, or perceived stress between the urban and rural adults before or during the COVID-19 period. However, alcohol consumption significantly decreased during the COVID-19 period compared to the pre-COVID-19 period in both urban and rural populations (p= 0.005 and < 0.001, respectively). In addition, both BMI and WC significantly increased during the COVID-19 period compared to the pre-pandemic era in urban areas (both p< 0.001), while in rural regions, only WC showed a significant increase (p= 0.040).

In urban areas, a significant increase was noted in the proportion of individuals experiencing food sufficiency during the COVID-19 era compared to the period before the pandemic. However, in rural areas, the food sufficiency status showed no significant change.

### Odds ratios and interactions of food sufficiency based on socio-demographic factors by region

Table 2 presents the ORs for perceived food sufficiency based on socio-demographic factors in urban and rural settings. In urban areas, the odds of reporting food sufficiency during the COVID-19 period, as opposed to before, ranged from 1.20 to 1.52 times higher for subcategories based on sex, household income, and age (95% confidence interval [CI], 1.01 to 1.80; p< 0.05). Female respondents, individuals from high-income households, and adults aged 20-64 years were more likely than their counterparts—male participants, those from low-income households, and seniors aged 65 years and older—to perceive their food as both qualitatively and quantitatively sufficient. In rural areas, only female respondents demonstrated a significant increase in the likelihood of reporting food sufficiency during the COVID-19 period compared to the pre-COVID-19 era (OR, 1.42; 95% CI, 1.06 to 1.89; p< 0.05). However, the data revealed no significant differences in the interaction between area (urban or rural) and socio-demographic factors with regard to food sufficiency.

### Changes in nutrient intake as a percentage of Dietary Reference Intakes for Koreans or daily energy intake by region

Table 3 presents the changes in nutrient intake as a percentage of the KDRI or daily energy intake. In urban areas, compared to KDRI standards, intake levels of total energy, sodium, calcium, and iron significantly decreased during the COVID-19 period relative to the pre-COVID-19 era (p = 0.042 for calcium and p< 0.001 for the other nutrients). The percentage of energy derived from carbohydrates also declined, from 60.1% to 58.6% (p< 0.001). In contrast, the proportion of energy intake from protein, fat, and saturated fat, as well as the intake of vitamin E, significantly increased during the COVID-19 period in urban areas (p< 0.001 for each, except vitamin E at p= 0.009). Similar trends were observed in rural areas, where the intake levels of total energy and iron significantly decreased during the COVID-19 period (p= 0.002 for total energy and p< 0.001 for iron), while the proportion of daily energy intake from fat and saturated fat significantly increased (p< 0.001 and 0.003, respectively).

## DISCUSSION

This study investigated changes in food sufficiency between urban and rural areas before and during the COVID-19 pandemic to elucidate the relationship between these changes and socio-demographic factors. Data from the 7th and 8th KNHANES were utilized to understand the changes before and during the COVID19 pandemic, with 2018 and 2019 serving as representative years of the pre-COVID-19 period and 2020 and 2021 as years during the pandemic.

Before this study began, it was hypothesized that food sufficiency had significantly deteriorated due to COVID-19. However, the analysis yielded contradictory results. In urban areas, the proportion of individuals experiencing food sufficiency significantly increased across all subgroups (sex, household income, and age) during the COVID-19 period compared to before the pandemic onset. These unexpected findings suggest a reevaluation of food sufficiency in urban areas during the COVID-19 period. A previous study by Jo et al. [25] revealed that adults in Seoul exhibited varying patterns of food security status and dietary habits based on sex, age, and income level during the COVID-19 period. The high-income group most frequently indicated an increase in ordering delivery and takeout meals. In contrast, affirmative responses to this item were less common among the low-income and older age groups. Additionally, the lowest reported increase in the rate of cooking and eating meals at home was observed among respondents from low-income backgrounds, older age groups, and male respondents. These results suggest that the availability of delivery and takeout services in urban areas may have provided access to a variety of food options. They also indicate that socioeconomic factors such as sex, age, and household income may play a crucial role in determining food sufficiency during the COVID-19 period.

Before the onset of COVID-19, urban areas exhibited a higher level of food sufficiency compared to rural areas [26]. The present findings indicate that during the COVID-19 period, the ORs for food sufficiency in urban areas were higher across all demographic subgroups, both quantitatively and qualitatively, than those recorded before the pandemic onset. In rural areas, although no significant changes in overall food sufficiency were observed during the COVID-19 period, a notable exception was observed among female, among whom food sufficiency significantly increased. A study from the United States identified a correlation between reduced involvement in meal preparation and decreased food sufficiency [27]. Furthermore, females in Korea’s rural areas are generally more engaged in meal preparation than their urban counterparts [28]. Given these varied findings, the significant increase in food sufficiency observed among rural female warrants further investigation into how different meal-related factors relate to food sufficiency. However, due to the scarcity of direct research connecting meal preparation behaviors among females and food sufficiency in rural settings, drawing definitive conclusions at this stage would be premature. Therefore, future research should delve more deeply into the impact of factors such as regional differences, sex, and meal preparation on food sufficiency. When analyzing the interaction between area (urban or rural) and various sociodemographic factors concerning changes in food sufficiency during the COVID-19 period, no significant results were found. Despite the lack of confirmed interactions, the observation that food sufficiency did not significantly improve in rural areas during the pandemic, in contrast to urban areas, underscores the importance of developing targeted food sufficiency policies for rural regions in the context of future pandemic preparedness.

To provide reliable evidence for future policies addressing food sufficiency in response to pandemic situations, this study examined changes in smoking status, household income, education level, participation in aerobic exercise, perceived stress level, and nutritional intake among adults aged 20 years and older in urban and rural areas before and during the COVID-19 period. The results indicated no significant differences in these variables between the pre-COVID-19 and COVID-19 eras in urban or rural settings. However, alcohol consumption decreased significantly during the COVID-19 period in both regions. Meta-analyses conducted in Europe and North America also reported a reduction in alcohol consumption during the COVID-19 period compared to before the pandemic began [29,30]. Similarly, the frequency of alcohol consumption among the Korean adult population generally declined during the COVID-19 period. Nevertheless, a paradoxical increase in alcohol consumption has been observed among individuals experiencing economic distress [31]. The observed decrease in alcohol consumption in urban and rural areas during the COVID-19 period in this study could be attributed to reduced social activities due to social distancing measures. Nonetheless, alcohol consumption patterns may vary based on income levels and social positions, suggesting that future research should consider socioeconomic factors in the analysis.

The rate of participation in aerobic exercise was anticipated to decline in both urban and rural areas due to the COVID-19 pandemic. The research findings indicated a trend toward reduced engagement in aerobic exercise during the pandemic compared to the period before the outbreak. However, this trend did not show a significant difference between the 2 periods within each area. Studies from Italy and Spain reported a decrease in physical activity during the COVID-19 period [32], which may have been impacted by the different responses and policies implemented by various countries. Korea’s social distancing policies were more lenient than those in European and North American countries. Furthermore, given the significant rise in BMI and WC in urban areas, along with the notable increase in WC in rural areas, it is essential to understand the shifts in health behaviors before and during the COVID-19 pandemic while considering the response policies of each country. To support future pandemic preparedness, new policies should promote aerobic exercise while adhering to social distancing guidelines. While these results do not establish a direct link with food sufficiency during the COVID-19 period, they can provide valuable insights for the formulation of food sufficiency policies in anticipation of future pandemics.

In this study, the nutritional intake survey was employed to analyze changes in nutrient consumption among urban and rural residents during the COVID-19 pandemic. This analysis was based on the KDRI standards for daily energy intake and compared nutrient consumption to the period before the COVID-19 outbreak. The results may also inform potential avenues for future research on the impact of these changes on dietary habits and food consumption patterns. During the COVID-19 period, both urban and rural areas displayed significant decreases in compliance with the KDRIs for total energy and iron intake. In urban areas, intake levels of carbohydrates, sodium, and calcium also decreased significantly during the pandemic. This reduction may be attributed to decreased physical activity resulting from social distancing measures implemented to control the spread of the virus [33], leading to a reduction in nutrient intake. However, in urban areas, the proportion of daily energy intake from protein increased significantly during the COVID-19 period compared to the pre-pandemic era. This increase could be linked to a significant rise in the purchase of processed meats, including meat, seafood, tofu, legumes, ham, sausages, and bacon, during the pandemic compared to before its onset [34]. Nonetheless, since the amount spent on food purchases does not directly indicate actual food consumption, future research should more thoroughly investigate the relationship between nutrient intake and consumption by food group before and during the COVID-19 outbreak.

Both urban and rural areas exhibited significant increases in the intake of fat and saturated fat relative to daily energy intake during the COVID-19 period. This rise may be linked to the heightened use of food delivery services for items like chicken, pizza, and burgers during the pandemic compared to the pre-COVID-19 era [35]. Furthermore, the increased consumption of nutritional supplements during the COVID-19 period [36] could explain the significant increase in vitamin E intake. However, given that this study presents cross-sectional data, the capacity to determine direct causation and outcomes is limited.

The KNHANES data used in this study explored both “perceived” food sufficiency and the “actual intake” of nutrients among participants. Examining these measurement variables together can provide mutually complementary information. Notably, this study revealed that although food sufficiency in urban areas has improved since before the COVID-19 pandemic, intake levels of calcium and iron have significantly decreased. These findings suggest that when developing food sufficiency policies for future pandemic preparedness, it is essential to consider not just the perception of food sufficiency but also actual nutritional intake.

This cross-sectional study utilized KNHANES data to explore changes and disparities in food sufficiency between urban and rural areas during the COVID-19 pandemic. The research was designed to comprehensively examine the patterns of food sufficiency changes during the pandemic, considering socio-demographic characteristics. However, establishing causality is not feasible within this study’s scope. Consequently, future research should incorporate methodologies such as longitudinal or cohort designs to more accurately discern the determinants of pandemic-related shifts in food sufficiency. Despite these constraints, the present study represents the first analysis of food sufficiency trends before and after the emergence of COVID-19 in urban and rural areas of Korea, incorporating detailed socio-demographic variables. The findings indicate that food sufficiency levels varied in association with socio-demographic factors during the pandemic in both settings. These results may represent a key evidence base for predicting food sufficiency trends in the context of pandemic preparedness, with consideration of regional socio-demographic influences. Furthermore, the study underscores the necessity for policy interventions tailored to food sufficiency challenges that may arise in rural communities during future pandemic situations.

## Figures and Tables

**Figure f1-epih-46-e2024045:**
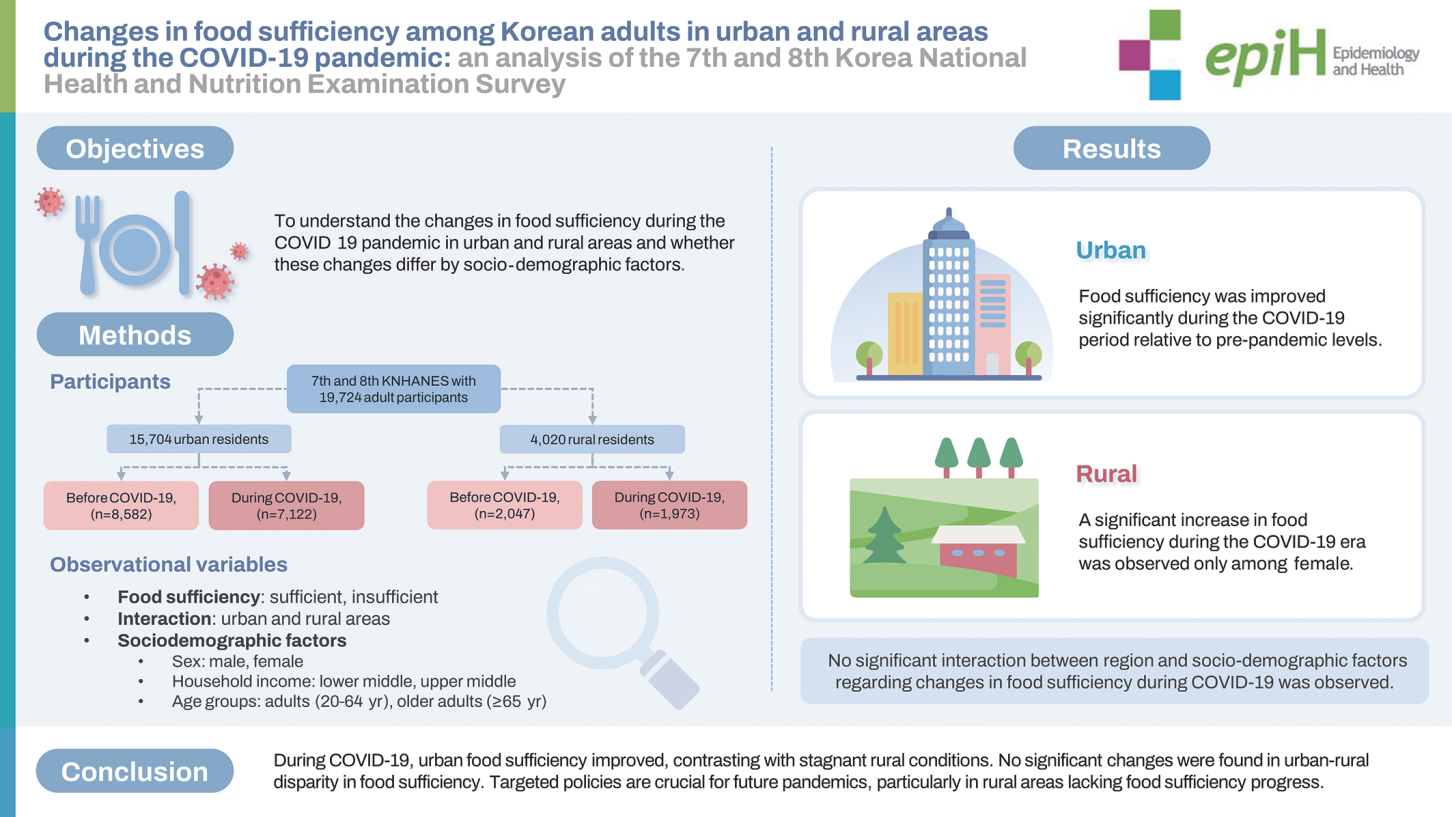


**Table 1.** General characteristics and food sufficiency status of participants by residential area

Variables	Total participants (≥20 yr, n=19,724)					
	Urban (n=15,704)			Rural (n=4,020)		
	Before COVID-19 (n=8,582)^1^	During COVID-19 (n=7,122)^2^	p-value^3^	Before COVID-19 (n=2,047)^1^	During COVID-19 (n=1,973)^2^	p-value^3^
Characteristics						
Age (yr)	47.4±0.3	47.8±0.4	0.355	54.1±1.2	56.4±1.2	0.196
Male/Female	6,646 (49.4)/9,058 (50.7)			1,759 (51.3)/2,261 (48.7)		
	3,599 (49.2)/4,983 (50.8)	3,047 (49.5)/4,075 (50.5)	0.728	893 (51.1)/1,154 (48.9)	866 (51.5)/1,107 (48.5)	0.825
Body mass index (kg/m^2^)	23.9±0.1	24.2±0.1	<0.001	24.3±0.1	24.3±0.1	0.926
Waist circumference (cm)	83.6±0.1	84.8±0.2	<0.001	85.0±0.3	85.8±0.3	0.040
Current smokers	2,198 (27.3)	1,870 (27.8)	0.328	491 (25.3)	447 (23.8)	0.347
Current drinkers	6,175 (76.8)	4,869 (74.1)	0.005	1,310 (70.6)	1,101 (61.7)	<0.001
Household income						
Lowest	1,464 (13.8)	1,215 (12.9)	0.350	629 (24.1)	649 (27.6)	0.548
Lower middle	2,106 (23.0)	1,675 (21.9)		563 (29.0)	486 (25.1)	
Upper middle	2,323 (28.6)	1,965 (29.9)		460 (25.3)	476 (26.4)	
Highest	2,667 (33.9)	2,241 (35.4)		385 (21.6)	347 (21.0)	
Education						
Elementary school or less	1,328 (11.3)	998 (9.6)	0.214	683 (27.0)	609 (26.5)	0.993
Middle school	736 (7.2)	627 (7.2)		249 (12.0)	238 (11.6)	
High school	2,695 (35.2)	2,270 (36.3)		547 (32.2)	492 (32.5)	
College or higher	3,449 (46.3)	2,748 (46.9)		446 (28.8)	454 (29.5)	
Participation in aerobic exercise	3,672 (47.4)	2,938 (46.4)	0.371	576 (33.9)	547 (33.2)	0.768
Feeling stressed	3,138 (40.3)	2,554 (39.4)	0.575	772 (42.6)	696 (40.8)	0.482
Food sufficiency status						
Sufficient	4,929 (57.4)	4,564 (64.1)	<0.001	1,094 (53.4)	1,172 (59.4)	0.069
Insufficient (quantity only or both quality and quantity)	3,653 (42.6)	2,558 (35.9)		953 (46.6)	801 (40.6)	

Values are presented as mean±standard error and number (%).

1 2018-2019.

2 2020-2021.

3 Continuous variables were subjected to t-tests with applied weighting to obtain p-values; Categorical variables were analyzed using the Rao-Scott chi-squared test, considering weighting to obtain p-values.

**Table 2.** Odds ratios^1^ for food sufficiency based on socio-demographic factors in urban and rural areas

Factors	Subgroups	Urban		Rural		p for interaction^2^
		Before COVID-19	During COVID-19	Before COVID-19	During COVID-19	
Sex	Male	1.00 (reference)	1.34 (1.14, 1.56)^***^	1.00 (reference)	1.17 (0.89, 1.54)	0.547
	Female	1.00 (reference)	1.43 (1.24, 1.65)^***^	1.00 (reference)	1.42 (1.06, 1.89)^*^	0.998
Household income	Lower middle	1.00 (reference)	1.20 (1.01, 1.43)^*^	1.00 (reference)	1.24 (0.91, 1.69)	0.689
	Upper middle	1.00 (reference)	1.52 (1.29, 1.80)^***^	1.00 (reference)	1.28 (0.89, 1.85)	0.423
Age (yr)	Adults (20-64)	1.00 (reference)	1.41 (1.22, 1.63)^***^	1.00 (reference)	1.26 (0.93, 1.70)	0.499
	Older adults (≥65)	1.00 (reference)	1.26 (1.04, 1.53)^*^	1.00 (reference)	1.33 (0.95, 1.87)	0.783

Values are presented as odds ratio (95% confidence interval). COVID-19, coronavirus disease 2019.

1 Using logistic regression models adjusted for sex, age, and household income.

2 The statistical significance of the interaction terms between residential area (rural or urban) and socio-demographic factors.

* p<0.05,

*** p<0.001.

**Table 3.** Changes in nutrient intake compared to KDRIs or daily energy intake by region

Nutrition intake^1^	Total participants (n=19,724)					
	Urban (n=15,704)			Rural (n=4,020)		
	Before COVID-19 (n=8,582)	During COVID-19 (n=7,122)	p-value^2^	Before COVID-19 (n=2,047)	During COVID-19 (n=1,973)	p-value^2^
Total energy intake	93.5±0.5	90.4±0.6	<0.001	96.1±1.2	90.6±1.1	0.002
Carbohydrates	60.1±0.2	58.6±0.2	<0.001	63.5±0.6	62.9±0.7	0.061
Protein	14.8±0.1	15.2±0.1	<0.001	14.2±0.2	14.5±0.2	0.056
Fat	20.1±0.1	22.0±0.1	<0.001	17.2±0.3	19.0±0.3	<0.001
Saturated fat	6.4±0.1	6.9±0.1	<0.001	5.3±0.1	5.8±0.1	0.003
Sodium	234.0±1.8	223.3±2.0	<0.001	238.9±5.2	237.1±3.4	0.763
Fiber	105.6±0.9	107.2±0.8	0.181	111.1±2.1	114.1±2.1	0.298
Calcium	83.7±0.8	81.5±0.8	0.042	80.7±1.6	78.5±1.4	0.294
Iron	141.5±1.5	127.8±1.5	<0.001	144.4±2.5	129.1±3.0	<0.001
Vitamin C	84.5±2.0	87.4±2.2	0.340	82.9±3.1	80.5±3.9	0.678
Vitamin E	54.9±0.5	56.7±0.5	0.009	52.8±1.2	53.6±0.9	0.598

Values are presented as % of intake amount compared to various reference points. COVID-19, coronavirus disease 2019; KDRIs, Dietary Reference Intakes for Koreans.

1 The reference value is the Estimated Average Requirement for total energy intake, calcium, iron and vitamin C; For macronutrients, the percentage of daily energy intake coming from carbohydrates, protein, fat and saturated fat were estimated; Adequate Intake data are used as reference points for sodium, fiber, and vitamin E.

2 From a linear regression analysis using a complex sample survey design; All models were adjusted for age, sex, and household income.
